# Utility of P Wave Indices on Electrocardiography in Predicting the Left Atrial Volume Index in Chronic Kidney Disease Patients

**DOI:** 10.7759/cureus.77809

**Published:** 2025-01-22

**Authors:** Umesh Kumar Pandey, Nikhil Chaudhary, Shivendra Singh

**Affiliations:** 1 Cardiology, Institute of Medical Sciences Banaras Hindu University (IMS-BHU), Varanasi, IND; 2 Nephrology, Institute of Medical Sciences Banaras Hindu University (IMS-BHU), Varanasi, IND

**Keywords:** ckd, lavi, left atrial volume index, p wave parameters, p wave peak time

## Abstract

Introduction: Left atrial volume (LAV) is a long-term marker for an array of physiological symptoms that can lead to high end-diastolic pressures - likening it to the echocardiographic equivalent of glycosylated hemoglobin (HbA1c). An increased left atrial volume index (LAVI) has been identified as an important biomarker for chronically increased LV diastolic pressure in chronic kidney disease (CKD) patients. We explored utilizing P wave parameters including newer parameters such as P wave peak time in predicting the increase in the LAVI in our study.

Material and methods: We included CKD patients (as per kidney disease: improving global outcomes (KIDGO)) with ages more than 18 years in our study from the nephrology department (OPD/IPD) at the Institute of Medical Sciences Banaras Hindu University (IMS-BHU), Varanasi, India. We excluded patients with rhythm abnormalities, decreased ejection fraction (<50%), valvular heart disease, coronary artery disease, and patients with poor echocardiographic assessment from our study.

Results: We enrolled 92 patients with CKD (as per KDIGO definition), out of which 72 met the inclusion-exclusion criteria. The study population's mean age was 53+/-11 years. Of the 72 individuals, 22 (30.6%) were females. Stage-wise depiction of CKD among the study population indicates that most patients (43, or 59.5%) were at stage 5. Thirty-six (50%) were on maintenance hemodialysis. Our analysis results indicate that the P wave peak time was the strongest predictor of the LAVI with a maximum area under the curve on the receiver operating characteristic (ROC) curve.

Conclusion: Our study suggests that electrocardiographic (ECG) parameters such as p wave peak time can predict LAVI in CKD and ECG is an easily available tool; thus, this could help stratify cardiovascular risk, especially left ventricular diastolic dysfunction.

## Introduction

Left atrial volume (LAV) is a long-term marker for an array of physiological symptoms that can lead to high diastolic pressures, likening it to an echocardiographic equivalent of glycosylated hemoglobin (HbA1c) [1]. Increased LAV size, as assessed through echocardiography, is strongly associated with mortality. Left ventricular diastolic dysfunction causes LAV enlargement [2], which is reflected in the LAV index (LAVI).

Chronic kidney disease (CKD) is associated with increased cardiovascular mortality and morbidity, and assessment of left ventricular diastolic dysfunction and LAV is important in predicting cardiovascular risk in CKD patients. [3] However, owing to the technical expertise required to perform echocardiography, this diagnostic tool is not readily available everywhere. In this study, we attempted to explore surrogate markers for LAVI on electrocardiography, mainly P wave indices, in patients with CKD.

## Materials and methods

Methods

On the day a patient enrolled in the study, their demographic details and disease history were noted. Each patient’s height and weight were recorded, and their body surface area was calculated using the Du Bois method in m^2^. Subjects’ blood investigations were performed on the interdialytic day, at which time the patient also underwent electrocardiography and echocardiography. The study took place from February 2023 to May 2024.

Electrocardiography assessment

All patients underwent 12-lead electrocardiography, recorded at 25 mm/sec and with a voltage of 10 mm/mV. The printout was then photographed with an Android smartphone. ECG results were assessed by a cardiologist, and the images were magnified when necessary. From limb lead II (D2), P wave dispersion (PWDis), maximum P wave duration (PWMax), and P wave peak time (PWPT) were calculated in milliseconds. Terminal P force (PTFV1) was calculated from lead V1. PWDis was calculated by subtracting the minimum P wave duration from the maximum P wave duration in lead D2. P wave peak time was measured as the duration between the beginning and peak of P waves in lead D2, and terminal P force from lead V1 was calculated by multiplying the depth of the negative portion of each P wave by its width, expressed in mV x msec.

Echocardiography assessment

Transthoracic echocardiography was performed by a cardiologist on a Sonosite Edge II echocardiography machine. Standard echocardiographic imaging was performed. The ejection fraction was calculated via the modified Simpson’s biplane method. LAV measurement was done at the end of the systole using the biplane disc summation method and indexed by body surface area to calculate LAVI in mL/m^2^. LAVI is left atrial volume indexed to body surface area and is considered normal at < 28 mL/m^2^ and increased at LAVI > 28 mL/m^2^.

## Results

Baseline characteristics

The study initially enrolled 92 patients with CKD (as per the KDIGO definition), of which 72 met the inclusion and exclusion criteria. The study population’s mean age was 53+/−11 years. Of the 72 subjects, 22 (30.6%) were female. Stage-wise depiction of CKD among the study population can be seen in Table 1, with most patients (43, or 59.5%) at stage 5. Furthermore, 36 (50%) of all participants were on maintenance hemodialysis. The etiology of patients’ CKD is depicted in Table 2, with diabetic kidney disease as the most common (39 patients, or 54.2%). Two subjects had a congenital anomaly of the kidney and urinary tract. In a presumed chronic glomerulonephritis biopsy, focal segmental glomerulosclerosis, IgA nephropathy, and lupus nephritis were each present in one subject. Of patients with chronic tubulointerstitial nephritis, one had polycystic kidney disease, and four had a history of nonsteroidal anti-inflammatory drug (NSAID) abuse. In the study population, 67 (93.1%) patients were hypertensive, 69 (95.83%) had anemia, and 14 (19.4%) were severely anemic. Additionally, 27 patients (37.5%) had volume overload.

**Table 1:** Distribution of the study population according to the CKD stage based on eGFR(creat) The data have been represented as the number of patients in each stage and the percentage of the total population (N=72).

CKD stage	Number	Percentage	
1	1		1.4
2	2		2.8
3A	1		1.4
3B	10		13.9
4	15		20.8
5	43		59.7

**Table 2:** Distribution of the population according to CKD etiology CGN = chronic glomerulonephritis, CTIN = chronic tubulointerstial nephritis, DKD = diabetic kidney disease, and HTN NS = hypertensive nephrosclerosis Data represent the number and percentage of cases of etiology within the total population (N=72).

Etiology	Number	Percentage	
CGN	24		33.3
CTIN	8		11.1
DKD	39		54.2
HTN NS	1		1.4

Results

In this study, we divided patients according to LAVI. Specifically, 52 patients were in the increased LAVI group, and 20 patients were in the normal LAVI group. The mean age in the increased LAVI group was 53.12+/−12.05 years. In the normal LAVI group, it was 51.10 +/−9.79 years. There was no significant statistical difference between the mean age of the two groups (p>0.05).

We compared P wave indices between the two groups.

**Table 3:** P wave indices compared between the two groups with increased and normal LAVI Data are represented as mean ± standard deviation. An independent sample T test was used, and p-values of <0.05 were taken as statistically significant.

P wave parameters	Group on the basis of LAVI	N	Mean	Std. Deviation	P-value
P WAVE DISPERSION (D2; in ms)	Normal	20	22.00	17.045	0.423
	Increase	52	25.38	15.525	
PFTV1 (in ms)	Normal	20	31.00	20.749	0.445
	Increase	52	34.23	17.640	
PW MAX(D2; in ms)	Normal	20	85.50	20.894	0.000
	Increase	52	108.27	21.848	
PWPT (D2; in ms)	Normal	20	27.00	7.327	0.000
	Increase	52	60.58	13.636	

Table 3 depicts the difference in mean P wave peak time. The value was 60.58+/−13.63 ms for the increased LAVI group, which is notably more than the value of 27+/−7.32 ms in the normal LAVI group. This difference was statistically significant (p=0.000). Similarly, the mean max P wave duration in lead II (PW max) was greater in the increased LAVI group (108.27+/−21.84 ms) than that in the normal LAVI group (85.50+/−20.89 ms), a statically significant difference (p=0.001). The other parameters, PFTV1 and P wave dispersion, had no statistically significant difference between the two groups. For the above test, an independent sample t-test was used, and p-values less than 0.05 were considered statistically significant.

We analyzed the correlation between LAVI and P wave indices, as depicted in Table 4.

**Table 4:** Pearson correlation coefficient with left atrial volume index (LAVI) Data depict the correlation between LAVI and P wave indices using the Pearson correlation coefficient, with p<0.05 considered statistically significant.

		P wave dispersion (D2) (in ms)	PFTV1 (in ms)	P wave max (D2)	PWPT ms
LAVI (in mL/m^2^)	Pearson Correlation Coefficient	0.121	0.044	0.533	0.752
	P-value	0.312	0.714	0.000	0.000

There was a significant positive correlation in LAVI with P wave peak time and max P wave duration only. With the other two parameters, P wave dispersion and terminal P force, there was no statistically significant correlation.

When we tested the electrocardiography parameters PWPT (D2), PW MAX (D2), PFTV1, and P wave dispersion against the echocardiographic parameter LAVI, we found that P wave peak time had strong predictor capacity with the maximum area under the curve (0.983 (p=0.00), 95% confidence interval: 0.961-1.0, followed by PW max 0.769 (p=0.000), 95% confidence interval: 0.657-0.881). P-values of <0.05 were taken as statistically significant (Table 5).

**Table 5:** Area under the curve of different electrocardiography parameters while testing against echocardiography parameter LAVI with a 28 mL/m2 cutoff

Test Result Variable(s)	Area Under the Curve	P-value	95% Confidence Interval
PWPT (D2; in ms)	0.983	0.000	0.961–1.0
PW MAX(D2; in ms)	0.769	0.000	0.657–0.881
PFTV1 (in ms)	0.576	0.318	0.422–0.731
P WAVE DISPERSION (D2; in ms)	0.579	0.300	0.434–0.724

Testing P wave peak time against a LAVI cutoff of 28 mL/m^2^ for increased LAVI on the receiver operating characteristic (ROC) curve, we found that a P wave peak time of 45 ms has a sensitivity of 84.6% and specificity reaching 100%. If we take the cutoff as 35 ms, sensitivity is 98.1%, and specificity is 85%. Meanwhile, the P wave maximum duration in lead D2 at 95 ms predicted increased LAVI with a sensitivity of 76.9% and a specificity of 60%.

## Discussion

Patients with CKD are at increased cardiovascular risk. CKD is strongly associated with impaired LV diastolic function [4]. Enlargement of the left atrium (LA) is associated with poor endpoints and may be useful for estimating cardiovascular events and death [5,6]. LAVI indirectly predicts long-standing diastolic dysfunction. Increased LAVI (LAVI > 28 mL/m^2^) is an independent predictor of heart failure, atrial fibrillation, ischemic stroke, and death, as well as a key structural change of diastolic heart failure, which can be used as an indicator for clinical evaluation of left ventricular diastolic function [7]. In a resource-constrained setting where echocardiography is not readily available, certain electrocardiographic parameters may act as surrogate markers to help identify patients with increased cardiovascular risk. This can facilitate the early diagnosis and treatment of at-risk patients.

In our study of 72 patients, we attempted to include patients with different stages of CKD, etiologies, and clinical profiles (e.g., with or without volume overload). We found that the electrocardiography parameter of P wave peak time correlates with LAVI, and a cutoff of 45 ms can predict increased LAVI. Maximum P wave duration also correlates, to some extent, with increased LAVI, but P wave dispersion and terminal P force are not strong predictors of LAVI. In comparison to the study done by Yildiz et al. [8] on hemodialysis patients, this study included the entire range of CKD stages, reemphasizing the significance of P wave peak time in predicting increased LAVI. Contrary to the aforementioned study’s findings, we found that P wave maximum duration may be influenced by LAVI to some extent.

This study also found that some P wave parameters, such as P wave dispersion and terminal P force in lead V1, do not meaningfully help predict LAVI. The reason for P wave dispersion not linearly correlating with LAVI might be the nature of this parameter, as increased PWD reflects prolongation of intra-atrial and interatrial conduction time with poorly coordinated conduction within the atrial muscles and discontinuous propagation of sinus impulses, mainly between the left and right atria. Beat-to-beat variation not only is a function of LA mechanical factors but is also affected by many other factors, such as athletic performance, hypertension, cardiac heart failure, diabetes, and hemodialysis, as well as other factors not relevant to this study, such as valvular heart disease and channelopathies [9-12]. These factors affect autonomic tone in patients, with inflammatory pathways affecting the conduction system.

Our study had certain limitations, including its small sample size and temporal changes in parameters. We used 2D echocardiography in the LV chamber assessment, which has limitations in image quality. Additionally, in the case of rhythm disorders and valvular defects, which independently affect P waves, this study’s results cannot be applied.

Despite these limitations, however, this study offers a novel method for determining cardiovascular risk in an economically inhibited and resource-constrained setting by using a simple tool: electrocardiography. This subject warrants further studies using better modalities to determine LV anatomy and testing with these P wave electrocardiographic parameters.

## Conclusions

Our study suggests that new electrocardiographic parameters, including P wave peak time, can independently predict LAVI in patients with CKD. Electrocardiography is an easily available tool, and point-of-care physicians can be trained to identify at-risk patients with it and promptly adjust management accordingly.

## References

[1] Lu ML, Lloyd MS (2022). The HbA1C of the heart: atrial volume index and outcomes of cardiac arrest. Resuscitation.

[2] Pritchett AM, Mahoney DW, Jacobsen SJ, Rodeheffer RJ, Karon BL, Redfield MM (2005). Diastolic dysfunction and left atrial volume: a population-based study. J Am Coll Cardiol.

[3] Zoccali C, Mallamaci F, Adamczak M (2023). Cardiovascular complications in chronic kidney disease: a review from the European renal and cardiovascular medicine working group of the European Renal Association. Cardiovasc Res.

[4] Pecoits-Filho R, Bucharles S, Barberato SH (2012). Diastolic heart failure in dialysis patients: mechanisms, diagnostic approach, and treatment. Semin Dial.

[5] Patel DA, Lavie CJ, Milani RV, Ventura HO (2011). Left atrial volume index predictive of mortality independent of left ventricular geometry in a large clinical cohort with preserved ejection fraction. Mayo Clin Proc.

[6] Barberato SH, Pecoits Filho R (2007). [Prognostic value of left atrial volume index in hemodialysis patients]. Arq Bras Cardiol.

[7] Mittal A, Punekar P (2020). Assessment of left atrial volume index in acute ischemic stroke patients; an observational study. J Assoc Physicians India.

[8] Yıldız İ, Özmen Yildiz P, Burak C (2020). P wave peak time for predicting an increased left atrial volume index in hemodialysis patients. Med Princ Pract.

[9] Ertem AG, Erdoğan M, Keleş T, Durmaz T, Bozkurt E (2015). P-wave dispersion and left ventricular diastolic dysfunction in hypertension. Anatol J Cardiol.

[10] Kim DH, Kim GC, Kim SH (2007). The relationship between the left atrial volume and the maximum P-wave and P-wave dispersion in patients with congestive heart failure. Yonsei Med J.

[11] Demir K, Avci A, Kaya Z (2016). Assessment of atrial electromechanical delay and P-wave dispersion in patients with type 2 diabetes mellitus. J Cardiol.

[12] Chen SC, Su HM, Huang JC (2015). Association of P-wave dispersion with overall and cardiovascular mortality in hemodialysis patients. Am J Nephrol.

